# Impaired Terminal Erythroid Maturation in β^0^-Thalassemia/HbE Patients with Different Clinical Severity

**DOI:** 10.3390/jcm11071755

**Published:** 2022-03-22

**Authors:** Thunwarat Suriyun, Pranee Winichagoon, Suthat Fucharoen, Orapan Sripichai

**Affiliations:** 1 Department of Biochemistry, Faculty of Medicine Siriraj Hospital, Mahidol University, Bangkok 10700, Thailand; mthunwarat.s@gmail.com; 2 Thalassemia Research Center, Institute of Molecular Biosciences, Mahidol University, Nakhonpathom 73170, Thailand; pranee.fuc@mahidol.ac.th (P.W.); suthat.fuc@mahidol.ac.th (S.F.); 3 National Institute of Health, Department of Medical Sciences, Ministry of Public Health, Nonthaburi 11000, Thailand

**Keywords:** β-thalassemia, hemoglobin, reticulocyte, mitochondria, erythropoiesis

## Abstract

Anemia in β-thalassemia is associated with ineffective erythropoiesis and a shortened lifespan of erythroid cells. The limited differentiation of β-thalassemic erythroblasts has been documented, but the characteristic feature of terminal erythroid maturation and its physiological relevance are not clearly described in β-thalassemias. Here, the red blood cell and reticulocyte cellular characteristics were determined in patients with β^0^-thalassemia/HbE in comparison to patients with iron deficiency anemia and healthy normal subjects. Severely affected β^0^-thalassemia/HbE patients showed the highest increase in immature reticulocytes, but the number of total erythrocytes was the lowest. Despite similar ranges of hemoglobin levels, β^0^-thalassemia/HbE patients had a higher number of reticulocytes and a greater proportion of immature fraction than patients with iron deficiency anemia did. In vitro CD34^+^ hematopoietic progenitor cells’ culture and flow cytometry analysis were conducted to investigate the erythroid maturation and mitochondrial clearance in β^0^-thalassemia/HbE erythroid cells as compared to normal cells. The delayed erythroid maturation and evidence of impaired mitochondria clearance were observed in β^0^-thalassemia/HbE cells at the terminal stage of differentiation. Additionally, increased transcript levels of genes related to erythroid mitophagy, BNIP3L and PINK1, were revealed in β^0^-thalassemia/HbE erythroblasts. The findings indicate that the erythroid maturation is physiologically relevant, and that the restoration of terminal maturation represents a potential therapeutic target for β-thalassemias.

## 1. Introduction

During erythropoiesis, multiple sequential steps are involved in the erythroid progenitor development and differentiation into mature red blood cells (RBCs), from progressive hemoglobinization, cell size reduction, and nuclear chromatin condensation followed by late-stage erythroblast enucleation, which produces immature erythrocytes (reticulocytes). Reticulocytes are then released from the bone marrow and other hematopoietic organs into blood circulation. The maturation of reticulocytes into RBCs only lasts a few days, involves completing the synthesis of hemoglobin (Hb), extensive remodeling of the plasma membrane and the selective removal of unnecessary organelles [1]. Mitochondrial elimination in erythrocytes is essential for the prevention of oxidative damage and erythrocyte destruction [2]. In various forms of anemia, such as hemoglobinopathies and iron deficiency anemia (IDA), the number of reticulocytes and the immature reticulocyte fraction (IMF) in the bloodstream increase due to an increased release of immature reticulocytes from the bone marrow in response to elevated erythropoietic stimulation [3,4].

The coinheritance of β-thalassemia and hemoglobin E (HbE, *HBB*: c.79G > A; the most common structural hemoglobin variant globally) makes β-thalassemia/HbE disease the most common thalassemia syndrome in southeast Asia. It has become a worldwide health problem due to increased population migration, comprising approximately 50% of the clinically severe β-thalassemia disorders [5]. Patients have a broad spectrum of clinical severity, including age of presentation, a requirement for transfusions, growth development and other complications [5,6,7]. An absent production of β-globin chains due to β^0^-thalassemia mutations consequently reduces cellular Hb synthesis in patients. Whereas HbE acts as a β^+^-thalassemia because the synthesis of the β^E^-globin chain is reduced due to the GAG to AAG mutation, in the triplet codon, 26 of the β-globin genes activate a cryptic splice site [8]. The mechanism(s) underlying the pathophysiology of the anemia is related to low hemoglobin production and imbalanced α- to non-α-globin chains synthesis, which constitutes an initial cause of ineffective erythropoiesis and destruction of erythroid cells [9]. The limited differentiation of β-thalassemic erythroblasts has been documented [10], but the terminal erythroid maturation is not clearly described. Although the maturation of reticulocytes into RBCs only lasts a few days, it directly affects the quality and quantity of the RBCs in circulation. In the present study, the reticulocyte cellular characteristics and its physiological relevance were determined in β^0^-thalassemia/HbE disease.

## 2. Materials and Methods

### 2.1. Subjects

A total of 201 Thai subjects including 90 β^0^-thalassemia/HbE patients, 21 IDA patients and 90 healthy normal control were recruited for the determination of complete blood cell count. β^0^-Thalassemia/HbE patients were recruited from the β-thalassemia/HbE cohort described previously [7], including non-splenectomized patients with different degrees of anemia, but excluding patients who recently (within 2 months) have received blood transfusion. IDA was classified by a low serum ferritin (<30 ng/mL) and a low Hb concentration (<11.0 g/dL for females and <12.5 g/dL for males according to the World Health Organization guidance) [11,12]. Clinical data were obtained from the patient medical records and physical examination.

### 2.2. Hematologic Studies

Complete blood counts including reticulocyte parameters were determined using an automated blood cell analyzer (ADVIA 120, Bayer, NY, USA). The Hb measurement was determined by the standard cyanmethemoglobin colorimetric method [13]. Detection was achieved with flow cytometry based on low- and high-angle laser light scatter signals that calculate the cellular volume and Hb concentration of individual cells [14]. Automated reticulocyte counting was based on the staining of RNA content using oxazine 750. Reticulocytes were further classified into three categories by maturity according to fluorescence intensity: low fluorescence ratio (L-Retic); medium fluorescence ratio (M-Retic); and high fluorescence ratio (H-Retic). Immature reticulocytes fraction (IRF = H-Retic + M-Retic) was used for evaluating erythropoietic activity [15].

### 2.3. In Vitro Culture of CD34^+^ Progenitor Cells

CD34^+^ hematopoietic progenitor cells were separated from peripheral blood by using the anti-CD34 magnetic microbeads positive selection kit (Miltenyi Biotech, Auburn, CA, USA) according to the manufacturer’s instruction. Cells were cultured in the three-phase erythropoietic culture system at 37 °C in 5% CO_2_ for 16 days. Culture protocol was a modification of the three-stage technique described by Douay L and Giarratana MC [16]. A basal media is the Iscove’s Modified Dulbecco’s Medium (IMDM; GIBCO, Grand Island, NY, USA) containing 20% fetal bovine serum (FBS; Sigma-Aldrich, St Louis, MO, USA), 3% human AB serum (Sigma-Aldrich), 10 ug/mL insulin (Sigma-Aldrich), 3 U/mL heparin (Sigma-Aldrich), 100 U/mL penicillin-streptomycin (GIBCO) and 200 ng/mL human holo-transferrin (PromoCell, Heidelberg, Germany). In the first phase (days 0–7), the basal media was added with 1 ng/mL interleukin-3 (IL3; Cell Signaling Technologies, Beverly, MA, USA), 10 ng/mL stem cell factor (SCF; Cell Signaling Technologies) and 3 U/mL erythropoietin (EPO; CILAG GmbH, Zug, Switzerland); the second phase (days 8–11) included the addition of 10 ng/mL SCF and 3 U/mL EPO, while in the third phase (days 12–16) 3 U/mL EPO and 300 ng/mL holo-transferrin were added.

### 2.4. Assessment of Erythroid Maturation

Cells were stained with phycoerythrin (PE)-conjugated antibody against transferrin receptor (CD71; BioLegend, San Diego, CA, USA) and allophycocyanin (APC)-conjugated antibody against glycophorin A (GPA; CD235a; BD Biosciences, San Diego, CA, USA) to investigate cell differentiation. In order to determine mitochondria content, cells were stained with MitoTracker Deep Red FM (Molecular Probes, Eugene, OR, USA) and co-stained with PE-conjugated antibody against CD71. Fluorescence signals were detected and analyzed using a BD FACSCalibur flow cytometer (BD Biosciences).

### 2.5. Quantitative RT-PCR Analysis

Total RNA was isolated from erythroid cells using TRIzol Reagent (Invitrogen, CA, USA) and treated with DNase I (Thermo Fisher Scientific, Waltham, MA, USA) following the manufacturer’s instructions. cDNA was generated using the RevertAid first strand cDNA synthesis kit (Thermo Fisher Scientific) with oligo-dT18 primer as per the manufacturer’s protocol. Quantitative RT-PCR assay for BNIP3L (BCL2 interacting protein 3 like) and PINK1 (PTEN induced kinase 1) expression was performed using the gene specific primers and the SYBR^®^ select master mix for CFX (Applied Biosystem, Foster City, CA, USA) in the CFX Connect™ Real-Time PCR machine (Bio-Rad Laboratories). The relative fold changes were calculated using the 2^ΔΔC (T)^ method by normalizing against ribosomal protein S18 (RPS18) expression.

### 2.6. Statistical Analysis

Statistical analyses were performed with PASW statistics (version 18.0; SPSS Inc., Chicago, IL, USA). The data are expressed as the mean ± standard deviation (SD) unless noted otherwise. Student’s *t*-test or two-way ANOVA multiple comparisons were used as appropriate. *p*-value of less than 0.05 was considered statistically different.

## 3. Results

### 3.1. Levels of Cellular Hb and Number of RBC in β^0^-Thalassemia/HbE Patients

The level of Hb is one of the criteria used for the β-thalassemia/HbE disease severity classification [7], thus, as expected, the significantly higher Hb levels in mild rather than severe patients were revealed (Hb = 7.9 ± 0.5 vs. 5.4 ± 0.7 g/dL, respectively). In general, defective Hb synthesis results in low RBC indices including small cells (low mean corpuscular volume, MCV) and low Hb content (low mean corpuscular hemoglobin, MCH, and mean corpuscular hemoglobin concentration, MCHC). An iron deficiency leads to the suppression of Hb synthesis and induces metabolic disorders. Table 1 shows the low RBC indices in β^0^-thalassemia/HbE and IDA patients. Interestingly, the number of RBC in mild β^0^-thalassemia/HbE was significantly higher than in the severe group (*p* = 8.2 × 10^−24^) and IDA patients, whereas the levels of MCH (the average Hb concentration within individual RBCs) were not different between these groups.

### 3.2. Heterogeneity of Reticulocytes in the Circulating Blood of Anemia Patients

The significant increases in the number of reticulocyte were observed in both β^0^-thalassemia/HbE and IDA compared to normal control (Table 1), but when comparing in the same Hb levels group, the β^0^-thalassemia/HbE patients had significantly higher reticulocyte counts and proportions of immature fraction than IDA patients did (*p* ≤ 10^−5^). Of note, at the similar ranges of Hb level (7.5–9.0 g/dL), the MCV levels of RBCs and reticulocytes in mild β^0^-thalassemia/HbE patients were significantly lower than in IDA patients (*p* = 4.3 × 10^−9^ and *p* = 1.8 × 10^−15^, respectively). However, this difference was not found in patients within the Hb range of 4.0–6.5 g/dL. The severe β^0^-thalassemia/HbE patients had the greatest induction in the percentage of reticulocytes in circulation (5.9 ± 1.3%) with a relative increase in the most immature fractions (H-Retic = 17.5 ± 6.0%), but the number of reticulocytes (cells/uL) was not significantly different between the severe and mild groups, suggesting loss of reticulocytes during its maturation into RBCs in the severe group. The marked decrease in levels of reticulocyte Hb (retHb) and increase in the rbcHb/retHb ratio were noticeable in the β^0^-thalassemia/HbE patients compared with IDA patients and normal control subjects. Although there was no difference of retHb levels in the mild and severe β^0^-thalassemia/HbE groups, a significantly higher rbcHb/retHb ratio in the mild group was found. Furthermore, the amount of Hb per unit volume of RBCs and reticulocytes were higher in the mild β^0^-thalassemia/HbE than in the severe groups (MCHC; *p* = 1.6 × 10^−10^ and CHCMr; *p* = 1.1 × 10^−8^, respectively).

### 3.3. Delayed Terminal Erythroid Maturation in β-Thalassemia/HbE

**Figure 1:**
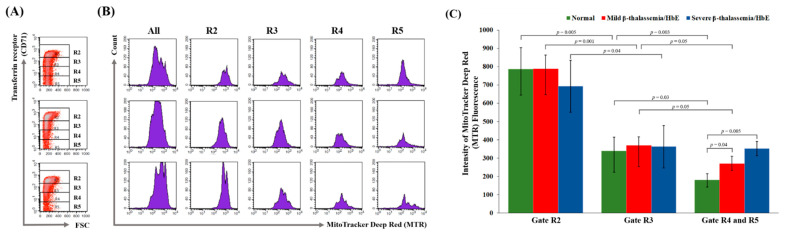
Flow cytometry analysis of in vitro culture erythroid cells at day 16. (**A**) Representative FACS plot of transferrin receptor (CD71) versus forward scatter (FSC) in erythroid cells’ culture of healthy normal control (upper panel), mild β^0^-thalassemia/HbE patient (middle panel) and severe β^0^-thalassemia/HbE patient (lower panel). The 10,000 cells per sample were analyzed (assigned as R1 population). The R2, R3, R4 and R5 cell populations were gated according to the fluorescent intensity of CD71. (**B**) Representative histogram plot of the MitoTracker Deep Red (MTR) stained erythrocytes from R1–R5 gates of healthy normal control (upper panel), mild β^0^-thalassemia/HbE patient (middle panel) and severe β^0^-thalassemia/HbE patient (lower panel). (**C**) Quantification of MTR fluorescence in healthy normal control (green bar), mild β^0^-thalassemia/HbE patient (red bar) and severe β^0^-thalassemia/HbE patient (blue bar), *n* = 3 in each group. Data are presented as mean ± SD. The two-tailed Student’s unpaired *t*-test was applied to compare the means between groups.

In this study, in vitro CD34^+^ progenitor cells culture was performed, then cells were harvested on day 16 of culture. At this stage, greater than 95% of cells showed positive CD235a staining (data not shown). For mitochondria determination study, cells were co-stained for CD71 and MTR mitochondria-specific dyes. Four subpopulations of erythroid cells were defined according to levels of CD71 expression that are known to decrease in level during erythroid maturation, as shown in the R2 to R5 gates (Figure 1A; R1 gate represents all cells). The R2 gate contained the cells in the early stage of differentiation with a larger size, whereas the cells in the R5 gate were of the late stage of erythroid maturation (Figure 1A compares cell size in the forward scatter (FSC) axis). The R4 and R5 populations became more abundant in cells derived from normal control, while these subpopulations were rare in cells derived from β^0^-thalassemia/HbE patients, indicating that terminal erythroid maturation was impaired in β-thalassemia/HbE cells. It is known that mitophagy aids erythroid maturation, and therefore, the intensity of MTR fluorescence reflecting the number of mitochondria was further analyzed in each cell subpopulation (Figure 1B). Staining with MTR alone provided optimal separation between stained and unstained cells (data not shown). The reduction of MTR fluorescent intensity in early to later erythroid subpopulations represents the loss of mitochondria during cell maturation. There was no difference in the intensity of MTR fluorescence in R4 and R5 subpopulations. Thus, these 2 gates were combined for further analysis. As expected, a gradual decline in MTR fluorescent intensity was revealed during differentiation of normal erythroblast cells (Figure 1C; R2 compared to R3 gates, *p* = 0.005; R3 compared to R4/5 gates, *p* = 0.03). The significant reduction of MTR fluorescent intensity in R2 to R3 subpopulation was also found in β^0^-thalassemia/HbE cells. However, the reduction of MTR fluorescent intensity in R3 to R4/5 subpopulations that represent the loss of mitochondria in terminal erythroid maturation was not observed in cells derived from severe β^0^-thalassemia/HbE patients. These findings reveal the defective mitochondria removal in the late-stage differentiation of β^0^-thalassemia/HbE erythroid cells.

**Figure 2:**
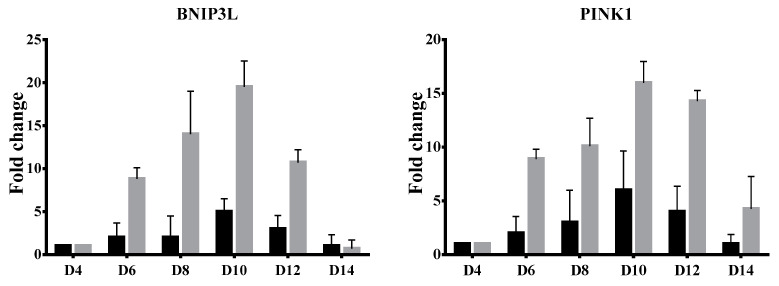
Quantitative RT-PCR analysis of BNIP3L and PINK1 transcripts. The mRNA levels were measured at days 4, 6, 8, 10, 12 and 14 of in vitro erythroid culture. Results from quantitative RT-PCR have been depicted as relative fold change of each day compared to day 4 with the mean and SEM value normalized to ribosomal protein S18 (RPS18) from three independent subjects of β^0^-thalassemia/HbE (gray bar) and healthy normal control (black bar).

Functional mitochondria contents were found to correlate with the expression patterns of mediators of mitophagy. In this study, sequential changes of the key mitophagy-specific genes BNIP3L and PINK1 were determined at various stages of erythroid differentiation using quantitative RT-PCR analysis. Figure 2 showed that BNIP3L and PINK1 were highly expressed in the early stage of terminal erythroid differentiation (day 10 of culture) and gradually downregulated with erythroblast maturation in both β^0^-thalassemia/HbE and normal cells. Additionally, levels of BNIP3L and PINK1 transcripts in β^0^-thalassemia/HbE were higher than in the control cells.

## 4. Discussion

The Hb concentration reported here was the actual Hb mass within the RBC (or cellular Hb), calculated from multiplying the cell volume with the Hb concentration on a cell-by-cell basis. The levels of Hb and number of RBCs in mild β^0^-thalassemia/HbE patients were significantly higher than severe patients. Generally, stress erythropoiesis induces fetal hemoglobin (HbF) production in β-thalassemia/HbE patients, but the levels of expression are highly variable [17]. These findings indicate that the low amount of cellular Hb, which is modified by the levels of HbE and HbF productions, and the lower number of RBCs in the circulation contribute to the severe clinical symptom in β^0^-thalassemia/HbE patients. Consistently, the number of circulating RBCs is modulated by the degree of ineffective erythropoiesis in the patients [10].

In the present study, significant heterogeneity in the reticulocyte population was clearly revealed in β^0^-thalassemia/HbE and IDA patients. Of note, the higher reticulocyte count and proportion of immature fraction were observed in β^0^-thalassemia/HbE patients. Despite the rise in immature reticulocytes in the circulation of the severe patients, no increase in the absolute reticulocyte counts was observed. Under basal erythropoiesis, reticulocytes remain in the bone marrow for 2–3 days before being released into the peripheral blood, and then transform into mature erythrocytes in 1–2 days [18,19]. In order to circulate through capillaries and respond to a range of shear stresses, mature RBCs must have the capacity to undergo substantial membrane deformation and shape changes. Specifically, it is known that membrane vesiculation leads to approximately a 20% loss of surface area [20] and that membranes of young reticulocytes are mechanically much less stable than those of mature cells [21]. As globular shapes confer lesser deformability than biconcave shapes, the presence of the biconcave-like geometries first detected in the low expression of the CD71 reticulocyte population contributes to its increased flexibility [22]. In addition to the shape effect, later stages of reticulocyte development also show significant increases to deformability and mechanical stability [23]. Accelerated erythropoiesis may result in the arrival of immature stress reticulocytes in peripheral blood, hence, the reticulocyte lifespan in the circulation increases to approximately 2–3 days during stress erythropoiesis [24]. An early release of immature reticulocytes with less flexibility and deformability from bone marrow into the blood circulation may lead to hemolysis of β^0^-thalassemia/HbE cells. Likewise, the apparent discrepancy between reticulocyte counts and IMF can be explained by neocytolysis and apoptosis of immature reticulocytes [25].

The analysis of peripheral blood suggested that the lower number of circulating RBCs in β^0^-thalassemia/HbE patients may partly result from defective terminal erythroid maturation. In mammalian erythroid cells, the expulsion of the nucleus followed by the removal of other organelles is necessary. Since approximately 30% of Hb is produced in reticulocytes, and heme is synthesized in the mitochondria, these organelles are among the last to be eliminated. The loading and unloading of oxygen by Hb can induce oxidant stress in RBCs [26]. Mitochondria are the major site for the production of reactive oxygen species (ROS) and can function as an apoptotic machinery [27]. Therefore, the timely elimination of mitochondria is essential for erythropoiesis and the survival of RBCs. The present study demonstrates the delayed terminal maturation and defective removal of mitochondria during β^0^-thalassemia/HbE erythroid maturation, which may contribute to the phenotype of patients. The mitochondria are eliminated through an autophagy-related process during terminal erythroid differentiation, and its defective process has been linked to anemia [28]. Increased mitophagy was previously reported in β^0^-thalassemia and β^0^-thalassemia/HbE erythroid [29,30].

The expression patterns of mediators of mitophagy correlate with mitochondria contents. BNIP3L/NIX induces mitochondrial autophagy in erythroid cells [31]. PINK1 is a key protein triggering mitophagy that increased expression with erythroblast maturation [32]. A reduced expression of Pink1 and Nix/Bnip3l, via disruption of sphingolipid signaling in a mice model, could lead to impaired mitochondria clearance and terminal erythropoiesis [32]. Here, sequential changes of BNIP3L and PINK1 were revealed in both β0-thalassemia/HbE and normal cells, but higher levels were found in patient cells. The induction of BNIP3L expression in β^0^-thalassemia/HbE erythroid cells was consistent with the previous report [29]. It is hypothesized that the pathology underlying β-thalassemia may contribute to mitochondrial damage, however, the relationship between mitophagy and abnormal hematopoiesis in β-thalassemia remains unknown. Thus, the delayed terminal erythroid maturation and defective removal of mitochondria in β^0^-thalassemia/HbE cells, despite the increased expression of BNIP3L and PINK1, reported here warrants further investigation. Insights into how mitochondria are eliminated in β-thalassemia/HbE erythroid cells should facilitate the development of novel therapeutic approaches for treating hematological disorders involving defective erythroid maturation.

Altogether, these findings indicate the association of delayed erythroid terminal maturation, reduced Hb synthesis in reticulocytes, a loss of immature reticulocytes, and fewer numbers of circulating RBCs with severely affected β^0^-thalassemia/HbE patients.

## Figures and Tables

**Table 1:** Distribution of red blood cell and reticulocyte parameters in β^0^-thalassemia/HbE patients, iron deficiency anemia patients and healthy normal control.

Characteristics	Normal	Hb 7.5–9.0 g/dL			Hb 4.0–6.5 g/dL		
		Iron Deficiency Anemia	Mild β-Thalassemia/HbE	*p*-Value *	Iron Deficiency Anemia	Severe β-Thalassemia/HbE	*p*-Value *
No. of cases	90	12	60		9	30	
Red blood cell							
Hb (g/dL)	13.4 ± 1.1	8.0 ± 0.9	7.9 ± 0.5	0.75	5.4 ± 0.8	5.4 ± 0.7 ^b^	0.87
RBC (×10^6^/uL)	4.6 ± 0.4	4.0 ± 0.5	4.2 ± 0.3	0.11	3.5 ± 0.6	3.1 ± 0.4 ^b^	0.03
MCV (fL)	89.5 ± 2.7	70.5 ± 7.2	60.3 ± 4.2	4.3 × 10^−9^	60.0 ± 8.9	60.2 ± 4.7	0.94
MCH (pg)	29.2 ± 1.4	19.9 ± 2.4	18.9 ± 1.7	0.09	15.7 ± 2.6	17.6 ± 1.6 ^a^	0.01
MCHC (g/dL)	32.7 ± 1.3	28.2 ± 1.6	31.3 ± 1.4	5.6 × 10^−9^	26.3 ± 3.5	29.2 ± 1.1 ^b^	3.5 × 10^−4^
Reticulocyte							
Retic (×10^3^/uL)	62 ± 21.1	118 ± 45.8	187 ± 41.0	1.8 × 10^−6^	101 ± 78.0	180 ± 39.3	1.4 × 10^−5^
retHb (g/dL)	0.6 ± 0.2	0.3 ± 0.1	0.1 ± 0.0	1.1 × 10^−10^	0.3 ± 0.2	0.1 ± 0.0	7.3 × 10^−6^
rbcHb/retHb ratio	25.2 ± 9.7	34.9 ± 13.0	70.9 ± 18.1	8.2 × 10^−9^	29.2 ± 27.4	48.2 ± 14.8 ^b^	9.4 × 10^−3^
Retic (%)	1.4 ± 0.4	3.0 ± 1.2	4.4 ± 1.0	1.6 × 10^−5^	2.9 ± 2.3	5.9 ± 1.3 ^b^	1.4 × 10^−5^
L-Retic (%)	93.8 ± 2.8	85.3 ± 4.6	75.8 ± 5.9	1.5 × 10^−6^	84.1 ± 6.8	64.0 ± 8.8 ^b^	2.2 × 10^−7^
M-Retic (%)	5.6 ± 2.7	12.9 ± 3.6	16.1 ± 3.0	1.4 × 10^−3^	12.3 ± 4.7	18.5 ± 3.4 ^b^	9.2 × 10^−5^
H-Retic (%)	0.6 ± 0.4	1.8 ± 1.3	8.1 ± 3.5	7.3 × 10^−8^	3.5 ± 2.5	17.5 ± 6.0 ^a^	6.1 × 10^−8^
IRF (%)	6.2 ± 2.8	14.7 ± 4.6	24.2 ± 5.9	1.5 × 10^−6^	15.9 ± 6.8	36.0 ± 8.7 ^b^	2.2 × 10^−7^
M/H ratio	11.6 ± 8.9	14.0 ± 15.0	2.3 ± 0.8	4.4 × 10^−8^	4.4 ± 1.9	1.1 ± 0.3 ^b^	3.5 × 10^−11^
MCVr (fL)	102.4 ± 3.5	97.7 ± 10.4	77.8 ± 5.0	1.8 × 10^−15^	79.4 ± 12.0	78.9 ± 4.9	0.83
CHr (pg)	32.4 ± 1.4	25.9 ± 3.9	20.8 ± 1.4	1.3 × 10^−11^	19.3 ± 3.2	20.0 ± 1.3	0.34
CHCMr (g/dL)	31.8 ± 1.3	26.5 ± 1.5	27.0 ± 1.1	0.18	24.5 ± 2.1	25.7 ± 0.7 ^b^	0.02

Hb, hemoglobin; RBC, red blood cell; MCV, mean corpuscular volume; MCH, mean corpuscular hemoglobin; MCHC, mean corpuscular hemoglobin concentration; Retic, reticulocyte; retHb, reticulocyte hemoglobin; rbcHb, mature red blood cell hemoglobin; L-Retic, low fluorescence ratio reticulocyte; M-Retic, medium fluorescence ratio reticulocyte; H-Retic, high fluorescence reticulocyte; IRF, immature reticulocyte fraction; MCVr, reticulocyte mean corpuscular volume; CHr, reticulocyte cell hemoglobin content; CHCMr, reticulocyte mean corpuscular hemoglobin concentration. Data are presented as mean ± s.d. * *p*-value of iron deficiency anemia group compared to β-thalassemia/HbE group in similar ranges of hemoglobin level. *p*-value of severe compared to mild β-thalassemia/HbE groups ^a^ 0.01–0.001; ^b^ <0.0001.

## Data Availability

Not applicable.
